# Sudden deafness in a patient with secondary syphilis and HIV infection

**DOI:** 10.1186/1471-2334-13-S1-P15

**Published:** 2013-12-16

**Authors:** Mădălina Gabriela Georgescu, Daniela Anghel, Dana Mihai

**Affiliations:** 1 Institute of Phono-Audiology and ENT Functional Surgery, Bucharest, Romania; 2 Carol Davila University of Medicine and Pharmacy, Bucharest, Romania; 3 Department of Neurology, Fundeni Clinical Institute, Bucharest, Romania; 4 Department of Dermatology, Fundeni Clinical Institute, Bucharest, Romania

## Background

We emphasize the importance of considering a diagnosis of early acquired syphilis in sexually active adults, and review the ENT manifestations and treatment of acquired syphilis.

## Case report

A 41-year-old man with left side hearing impairment since childhood presented with sudden hearing loss in the right ear; mild imbalance and clinical features suggestive of secondary syphilis (skin rash) developed lately. The first choice of treatment in the ENT department was followed after initial favorable evolution by relapsed sudden hearing loss in the right ear, dizziness, vertigo, nausea, and vomiting and gait impairment. The correct diagnosis was made after multiple evaluations in ENT, neurology, dermatology and infectious diseases departments.

Combined ENT and infectious diseases treatment resulted in only partial recovery of hearing in the right ear and complete absence of dizziness.

## Conclusion

With the exponential rise in syphilis cases in Romania, there has been a reemergence of presenting manifestations that had previously become rare. Early syphilis should be considered in all sexually active adults who present with deafness, as prompt diagnosis and treatment are crucial for maximum recovery.

